# A Study on the Bending Stiffness of Reinforced Concrete Tunnel Segments with Added Steel Fibers

**DOI:** 10.3390/ma18010048

**Published:** 2024-12-26

**Authors:** Fan Zhang, Wouter De Corte, Luc Taerwe, Weibiao Cao, Xian Liu

**Affiliations:** 1 College of Civil Engineering, Tongji University, Shanghai 200092, China; fanzhang@tongji.edu.cn (F.Z.); luc.taerwe@ugent.be (L.T.); 2 Department of Structural Engineering and Building Materials, Ghent University, 9052 Ghent, Belgium; wouter.decorte@ugent.be; 3 Shanghai Tunnel Engineering and Rail Transit Design & Research Institute, Shanghai 200235, China; cao.weibiao@stedi.com.cn; 4 State Key Laboratory for Hazard Reduction in Civil Engineering, Tongji University, Shanghai 200092, China

**Keywords:** steel-fiber-reinforced concrete, tunnel segments, full-scale tests, analytical model, segment stiffness

## Abstract

In recent years, steel-fiber-reinforced concrete (SFRC) has been increasingly applied in shield tunnel engineering. However, most research on SFRC segments focuses on the load-bearing capacity, while the tunnel deformation is an equally critical indicator that decides if the tunnel can operate safely during service conditions. Therefore, it is essential to also study the stiffness variations in SFRC segments, which is closely connected to the serviceability limit state (SLS). To investigate the influence of SFRC on segment stiffness, full-scale four-point bending tests and analytical calculations are carried out on both traditional reinforced concrete (RC) segments and SFRC segments with rebars. A C55 plain concrete is used in the RC segment, and for SFRC, 30 kg/m^3^ steel fibers are added. The segment stiffnesses are calculated and analyzed, and compared between test and analytical results. This study shows that the addition of steel fibers to traditional reinforced concrete segments can enhance the bending stiffness. This effect becomes apparent only after the segments crack. Initially, the effect is strong but then becomes weaker, with increasing load. The added 30 kg/m^3^ steel fibers generate a maximum of 33% in stiffness increment for a segment with 2.1% reinforcement. Further analysis indicates that the transfer of stresses in the cracked SFRC results in a stiffness improvement, but after cracking, the contribution of the reinforcement to the flexural resistance increases while the contribution of the SFRC gradually decreases. Thus, the effect is weak at high load levels. This paper contributes to a better understanding of the effect of SFRC on the stiffness of segments, as relevant for SLS requirements.

## 1. Introduction

In recent years, the applications of different types of fiber-reinforced concrete (FRC) in tunnel segments, such as steel-fiber-reinforced concrete (SFRC) [1,2,3,4], synthetic-fiber-reinforced concrete [5,6,7], basalt-fiber-reinforced concrete [8], have been widely studied, because FRC demonstrates outstanding impact resistance, crack control, and toughness [9], which can improve the structural properties. In particular, nowadays, some infrastructures show degradation after some decades because of cracks and corrosion, which can damage the bearing capacity [10,11,12] and stiffness [13] of concrete structures such as bridges and tunnel linings. This demonstrates the necessity of using FRC to optimize these structures. In practice, SFRC is widely used in tunnel engineering [14,15], and research [16] shows that it can make the segments more competitive, compared to traditional reinforced concrete (RC) segments, when considering the full life cycle cost.

The application of SFRC in shield tunnels can be divided into three categories according to the type of reinforcement [1]: (1) the bearing capacity of the SFRC is fully considered, and the traditional reinforcement is completely replaced, referred to as steel-fiber-reinforced concrete segments (SFRC segments); (2) the traditional reinforcement is partially replaced, referred to as reinforced-steel-fiber-reinforced concrete segments (RC-SFRC segments); (3) the contribution of the SFRC to the bearing capacity is not considered, and the fibers are only used to control crack width, and the traditional reinforcement is not replaced. Table 1 lists experimental research results for segments with SFRC applied in different ways. In the table, “Unkn” indicates information not provided in the literature, “+” indicates that the performance is better than that of a traditional RC segment, while “−” indicates an inferior performance, and “=” indicates an equal performance. No values means that there is no quantitative analysis in the reference.

As can be seen from Table 1, most research focuses on the bearing capacity. Using SFRC without rebar replacement results in a significant increase in load-bearing capacity (about 14%). Further, when SFRC partially replaces the steel bars according to some design considerations, the bearing capacity can at least remain equal. However, when all steel bars are replaced by SFRC, an improper use of SFRC (e.g., insufficient fiber content), will lead to a reduction in bearing capacity, particularly in terms of ultimate bearing capacity. Finally, Table 1 also reveals that there are very few studies that focus on the stiffness improvement. In reference [1], the stiffness is defined as the slope of the bending moment-deflection curve from the first cracking to the yielding of the steel bars. The definition of stiffness in this paper is addressed in Section 3.

This lack of experimental results related to bending stiffness is also found in the available analytical calculation methods. Instead, based on extensive experimental campaigns, analytical methods to calculate the bearing capacity of SFRC segments have been proposed in the literature. Based on the constitutive law of SFRC in the fib Model Code 2010 [22], Bin et al. [24] proposed a formula for the ultimate bearing capacity of SFRC segments, Lin et al. [25] proposed a design method for SFRC segments to ensure sufficient ductility and prevent brittle failure. Liu et al. [1], Bernardino et al. [26], and de la Fuente et al. [27], based on the constitutive law for SFRC by RILEM [28], proposed various formulas for the ultimate bearing capacity of SFRC segments. Additionally, several relevant standards [22,28,29,30,31] provide further calculation guidance. All of these methods consider that the concrete in the compression zone is in a plastic state and reaches ultimate strain, thus none of these methods can be used for stiffness evaluation. In contrast, Tiberti et al. [32] conducted a theoretical analysis of the bearing capacity of SFRC segments with different crack widths, based on a rigid-plastic model for SFRC, and addressed the serviceability state. However, this analytical method mostly focused on the bearing capacity, and experimental results from reference [5] indicate that the method has shortcomings.

Segment stiffness is an important parameter for shield tunnels in service. To ensure the safe operation of shield tunnels, the deformation of the tunnel is an important indicator for evaluating its safety status [33,34,35]. Since variations in segment stiffness will affect tunnel deformation, it is very important to understand the effect of SFRC on the segment stiffness.

Considering the current insufficient research on this matter, in this paper, full-scale four-point bending tests on RC segments and RC-SFRC segments are performed, and an analytical calculation method for the bending stiffness of SFRC segments is proposed. The derived analytical method is verified through experimental data, and a comparative analysis of the bending stiffness of both types of segments is performed. Through full-scale tests, this paper confirms the enhancement effect of SFRC on segment stiffness, and more importantly, analytical calculations reveal the mechanism behind this enhancement, and how the enhancement changes at different load levels. This paper contributes to a better understanding of the effect of SFRC on the stiffness of concrete tunnel segments, as relevant for SLS requirements, and the analytical model addresses the current deficiency in standards regarding the calculation methods for the bending stiffness of SFRC segments.

## 2. Experimental Program

### 2.1. Test Specimens

#### 2.1.1. Segment Size and Reinforcement

**Figure 1:**
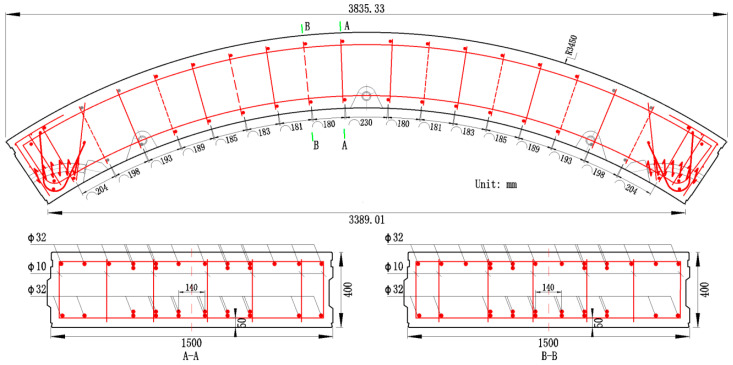
Segment dimensions and reinforcement.

The test segments are selected from a tunnel project in Hangzhou City, and meet the requirement of Code GB/T 22082-2017 [36]. The test segments have a width of 1500 mm, a thickness of 400 mm, and an outer diameter of 6900 mm, with a central angle of 67.5°. The main reinforcement at both the intrados and extrados consists of HRB400 steel bars, with a diameter of 32 mm, an elastic modulus of 200,000 MPa, and a guaranteed yield strength of 400 MPa. There are 16 bars at both the intrados and extrados, and the reinforcement ratio is 2.57%. To ensure sufficient spacing between adjacent reinforcement bars in the transverse direction, some of the rebars are arranged in two layers without a gap. There are 10 reinforcement bars in the first layer and 6 reinforcement bars in the second layer for the intrados, and 12 and 4 reinforcement bars in the first and second layer, respectively for the extrados. The thickness of the concrete cover is 50 mm on the longitudinal rebars. The segment dimensions and the reinforcement configuration are shown in Figure 1.

#### 2.1.2. Concrete Mixture Composition

A C55 concrete (*f*_c;cube_ ≥ 55 MPa) is used for the segment. The concrete mixture composition is shown in Table 2. The fiber content for the SFRC specimens is 30 kg/m^3^. In order to make the workability of the SFRC similar to that of the plain concrete (PC), the composition of SFRC is slightly adjusted.

Hook-end steel fibers are used in the tests, and the parameters are shown in Table 3.

#### 2.1.3. Manufacturing of the Test Specimens

In the experiments, one conventionally reinforced concrete segment and one segment reinforced with steel bars and steel-fiber-reinforced concrete were cast. During the casting of the segments, small test specimens, such as 150 mm cubes, 150 mm × 150 mm × 300 mm prismatic specimens, and 150 mm × 150 mm × 550 mm beam specimens, were cast with the same batch of concrete, to test the material properties such as the compressive strength, and the flexural strength. These specimens were compacted by a vibrating table and cured in a curing room for 28 days. The size and number of specimens are outlined in Table 4.

All test specimens were cast at a specialized segment production plant. Due to the extensive reinforcement of the segment, it was estimated that the ultimate bearing capacity of the segment could exceed the load capacity of the test reaction frame. Therefore, each segment was cut, in the longitudinal direction, into two equal specimens to fulfill the bearing capacity of the test setup, which means the width of the actual segment specimens is 750 mm. The four segment specimens were labeled as RCS1, RCS2, and RC-SFRCS1, RC-SFRCS2.

### 2.2. Test Program

#### 2.2.1. Material Tests

Compression tests were carried out for the cubic and prismatic specimens, according to the Chinese Code GB/T 50081-2019 [37]. Bending tests were carried out on the beam specimens, according to EN 14651 [38]. All test procedures adhered to the relevant standards, the details of which are omitted here for brevity. The test results are presented in Section 4.1.

#### 2.2.2. Segments Bending Tests

**Figure 2:**
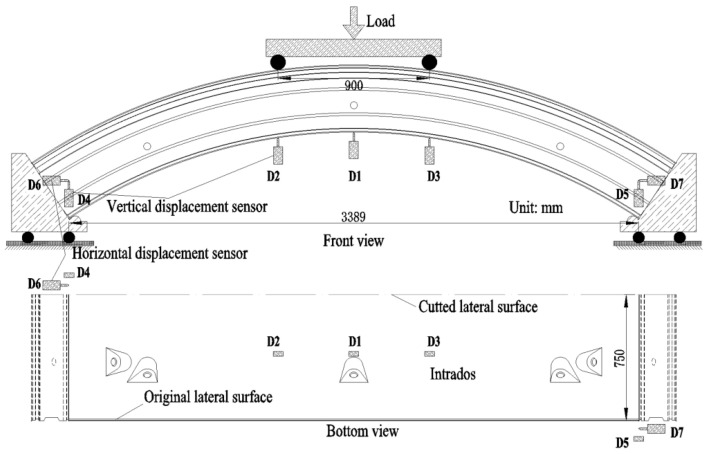
Loading and displacement sensors layout (D: displacement sensor).

Four-point bending tests were carried out on the segment specimens. The segments were put on two supports that can slide horizontally along rails (Figure 2). The load was applied on the distributing beam at the extrados by a hydraulic jack (made by Shanghai LianDi Industrial Development Co., Ltd., Shanghai, China) with a maximum capacity of 5000 kN. The distance between the two loading points was 900 mm. The internal rollers were positioned directly below the intrados corners of the segment. Since the external rollers did not bear any load (due to the support rotating around the internal roller) after the load was applied, the distance between the internal rollers is considered as the span of the support points.

The load was applied step by step, according to the loading steps given in Table 5. The next load step was applied only when the segment response was stable. Initially, due to the low hydraulic pressure, it was challenging to adjust the oil pressure to a specific load value. Therefore, the load values for the first four steps were determined from the data of the pressure sensor after each test, which could be a little different for each test.

**Figure 3:**
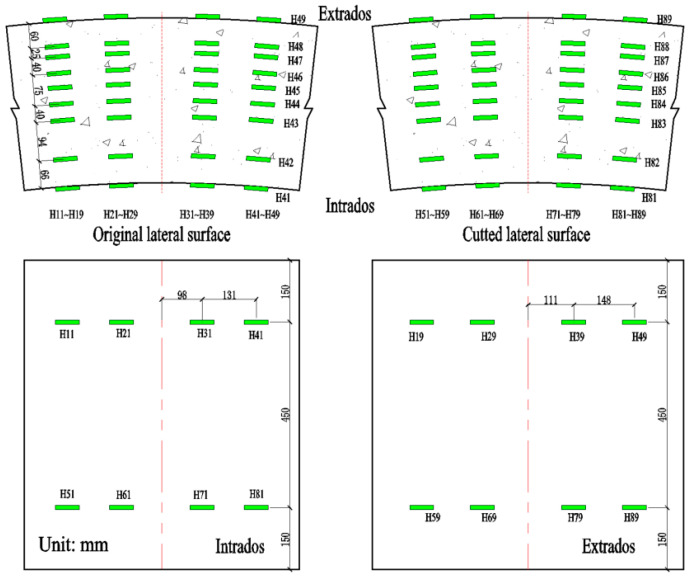
Layout of concrete strain gauges at lateral, intrados, and extrados surfaces (the green rectangle represents the strain gauge).

The load values, deformations and concrete strains were monitored during the experiment. The load value was measured through a pressure sensor connected to the hydraulic pump. The deformations were measured by 7 LVDTs (made by Liyang Instrument Factory, Changzhou, China). One LVDT was located at midspan (D1) and two LVDTs were located at the loading points (D2–D3), to measure the vertical displacements. Additionally, at diagonal positions at both ends of the segment supports, one horizontal and one vertical LVDT were installed to measure horizontal and vertical displacements (D4–D7). The layout of the LVDTs is illustrated in Figure 2. The concrete strains were measured through strain gauges (made byHuangyan Chengli Engineering Sensor Factory, Taizhou, China), located between the loading points. The strain gauges were positioned in four columns at each side of the segment. Each column contained nine gauges. The arrangement of the strain gauges is depicted in Figure 3. From bottom to top, the distances of the concrete strain gauges from the inner arc surface were 0 mm, 66 mm, 160 mm, 200 mm, 240 mm, 275 mm, 315 mm, 340 mm, and 400 mm, respectively.

## 3. Analytical Model for SFRC Segments

### 3.1. Basic Assumptions

To facilitate the analysis of the mechanical properties of SFRC segments, the following assumptions are made:

1. The deformation of the segments satisfies the plane section assumption.

2. The bond between steel reinforcement and concrete is assumed to be perfect, without slip.

These assumptions are crucial to simplify the analysis and focus on the basic mechanical behavior of SFRC segments under bending.

The model assumes perfect bond between the steel reinforcement and concrete, which limits the application of this analytical model, as it cannot be used for segments with poor bonding conditions of the rebars. Moreover, it is not suitable to evaluate crack widths, because slippage is the main contribution to the crack width. However, this limitation does not affect the macro-mechanical analysis, such as the stiffness.

Additionally, the effect of reinforcement confinement on concrete compressive strength and ductility is not considered. Reinforcement confinement can enhance both the strength and ductility of concrete [39,40]. However, the primary focus here is on studying the stiffness of segments, which is relevant to the serviceability limit state (SLS). In this context, the compressive stress is relatively low, and the effect of confinement can be disregarded.

### 3.2. Constitutive Laws

#### 3.2.1. Concrete in Tension

**Figure 4:**
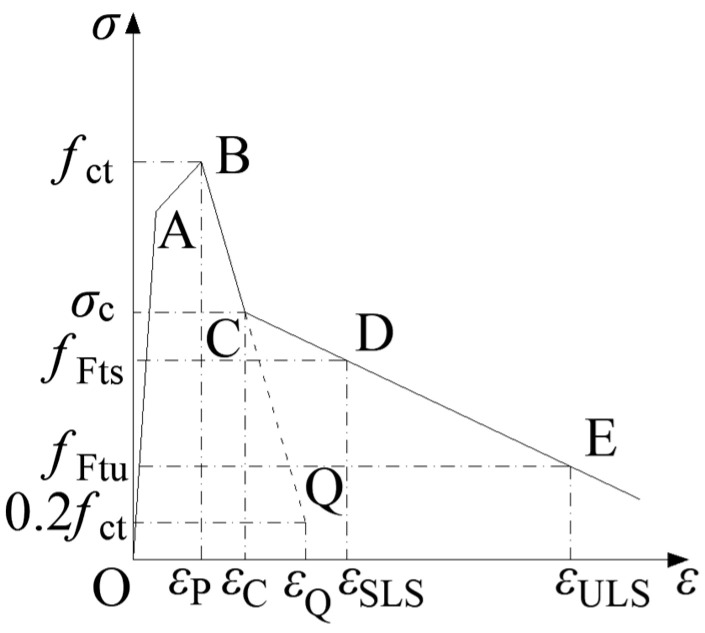
Schematic diagram of the constitutive law for concrete in tension according to.

The constitutive law for concrete in tension, provided in *fib* Model Code 2020 [23] and shown in Figure 4, is adopted for this analysis. The curve OABQ represents the constitutive law for PC, while the curve OABCDE represents the constitutive law for SFRC. There are several key points in the model. Point A represents the proportionality limit of concrete in tension. The stress at this point is 0.9 *f*_ct_ (where *f*_ct_ is the tensile strength of concrete) and the corresponding strain is calculated based on the elastic modulus. Point B represents the tensile strength of the concrete. The stress at this point is the tensile strength of concrete, and the strain *ε*_p_ is 0.00015. Point D corresponds to the residual strength at the SLS. The stress is the residual strength *f*_Fts_ and the strain is *ε*_SLS_ at SLS. Point E corresponds to the residual strength at the ultimate limit state (ULS). The stress is the residual strength *f*_Ftu_ and the strain is *ε*_ULS_ at ULS. Point Q represents the point where the strength of the plain concrete decreases to 0.2 *f*_ct_ after cracking, and the strain is *ε*_Q_. Point C is the intersection point between the lines BQ and DE. The values of each point in the laws are calculated according to Equations (1)–(7).

(1) $$\varepsilon_{Q}=G_{F}/f_{ct}l_{cs}+\varepsilon_{P}-0.8f_{ct}/E_{c}$$

(2) $$G_{F}=0.085f_{cm}^{0.18}$$

(3) $$f_{Fts}=0.37f_{R1}$$

(4) $$\varepsilon_{SLS}={CMOD}_{1}/l_{cs}$$

(5) $$f_{Ftu}=f_{Fts}-\frac{w_{u}}{{CMOD}_{3}}(f_{Fts}-0.57f_{R3}+0.26f_{R1})$$

(6) $$\varepsilon_{ULS}=\frac{w_{u}}{l_{cs}}=min(\varepsilon_{Fu},2.5/l_{cs})$$

(7) $$w_{u}=\varepsilon_{ULS}l_{cs}$$

In the equations, *G*_F_ (N/mm) represents the fracture energy of PC with a similar compressive strength as the SFRC. *f*_ct_ denotes the tensile strength of the concrete. *f*_cm_ is the average compressive strength of the concrete. *ε*_p_ is the cracking strain of the concrete, set at 0.00015. *f*_R1_ is the residual strength obtained from a three-point bending test according to EN 14651 [38], corresponding to the CMOD_1_ of 0.5 mm. *f*_R3_ is the residual strength when the CMOD_3_ is 2.5 mm. *ε*_Fu_ is the ultimate tensile strain of the SFRC. When the internal stress distribution across the section is uniform, it is taken as 1%. When the distribution is not uniform, it is taken as 2%, the value which is adopted in this paper. *l*_cs_ represents the characteristic length, it is the mean distance between cracks according to *fib* Model Code 2020. This distance ranges in the tests from 100 mm to 150 mm, and is taken as 125 mm in this analysis.

#### 3.2.2. Concrete in Compression

The influence of steel fibers on the compressive strength is limited, but the fibers can significantly enhance the ductility [41,42,43], and the compressive constitutive law for SFRC should reflect this difference. Currently, standards do not provide a specific compressive constitutive low for SFRC, but there are several laws proposed for SFRC in the literature [44,45,46,47], and the constitutive law proposed in [45] is adopted in the analysis, which includes the following equations (Equations (8)–(15)).

(8) $$\sigma =(1-d_{cf})E_{cf}\varepsilon$$

(9) $$d_{cf}=\left\{\begin{array}{c} 1-\frac{\rho_{cf}n}{n-1+x^{n}}\;\;\;\;\;\;\;\;\;\;\;\;\;\;\;\;\;\;\;\;\;\;\;\;\;\;(x\leq 1)\\ 1-\frac{\rho_{cf}}{\alpha_{cf}{\left(x-1\right)}^{2}+x}\;\;\;\;\;\;\;\;\;\;\;\;\;\;\;\;\;(x>1) \end{array}\right.$$

(10) $$\rho_{cf}=\frac{f_{cf,r}}{E_{cf}\varepsilon_{cf,r}}$$

(11) $$n=\frac{E_{cf}\varepsilon_{cf,r}}{E_{cf}\varepsilon_{cf,r}-f_{cf,r}}$$

(12) $$x=\frac{\varepsilon }{\varepsilon_{cf,r}}$$

(13) $$\alpha_{cf}=\left(0.157f_{cf,r}^{0.785}-0.905\right)[1-0.0192\left(\frac{l_{f}}{d_{f}}\right)V_{f}^{0.08}]$$

(14) $$\varepsilon_{cf,r}=(700+172\sqrt{f_{cf,r}})(1+0.189V_{f}\frac{l_{f}}{d_{f}})/10^{6}$$

(15) $$E_{cf}=\frac{10^{5}}{2.2+\frac{34.7}{f_{fcu}}}(1-0.006V_{f}\frac{l_{f}}{d_{f}})$$

In the equations, *E*_cf_ is the elastic modulus of SFRC. *f*_fcu_ is the compressive strength of SFRC tested from cubic specimens, *f*_cf,r_ is the compressive strength tested from the prismoid specimens, and *ε*_cf,r_ is the strain corresponding to *f*_cf,r_. *V*_f_, *l*_f_ and *d*_f_ are the fiber content, length and diameter, respectively.

The constitutive equations for SFRC are consistent with the format for the compressive behavior of PC as outlined in the Chinese Code GB 50010-2010 [48]. Therefore, the compressive law from this code is adopted here. The specific formulas can be referenced directly from the code, and are not repeated here for brevity.

#### 3.2.3. Steel Rebars

The reinforcement bars used in the segments typically exhibit a distinct yield point in tension as well as in compression. Therefore, the constitutive law for the reinforcement bars assumes a linear stress–strain relationship before yielding, and constant stress after yielding. The constitutive law is calculated according to Equations (16)–(18).

(16) $$\sigma_{s}=E_{s}\varepsilon_{s}■■■■■■■■■■■■■■■■■-f_{y}/E_{s}\leq \varepsilon_{s}\leq f_{y}/E_{s}$$

(17) $$\sigma_{s}=-f_{y}■■■■■■■■■■■■■■■■■\varepsilon_{s}\leq -f_{y}/E_{s}$$

(18) $$\sigma_{s}=f_{y}■■■■■■■■■■■■■■■■■\varepsilon_{s}\geq f_{y}/E_{s}$$

In the equations, *E*_s_ is the elastic modulus. And *f*_y_ is the yield strength.

### 3.3. Derivation of the Analytical Model

**Figure 5:**
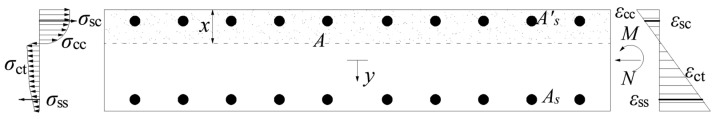
Strain and stress diagram of segment cross section (*ε*_sc_, *σ*_sc_: the strain and stress of rebars at the extrados; *ε*_ss_, *σ*_ss_: the strain and stress of rebars at the intrados; *ε*_cc_, *σ*_cc_: the compressive strain and stress of concrete; *ε*_ct_, *σ*_ct_: the tensile strain and stress of concrete; *x*: the height of compression zone; *A*, *A*_s_, *A’*_s_: the areas of the segment cross section, the rebars at intrados and extrados; *M*, *N*: the bending moment and axial force).

Based on the hypotheses and constitutive laws given in Section 3.1 and Section 3.2, the analysis method to calculate the mechanical properties of the segments subjected to bending can be derived through the following steps. When the segment is subjected to an axial force *N* and a bending moment *M*, the curvature due to deformation is *u*, and the height of the compression zone is *x*. According to the plane section assumption, the strain and stress diagrams of the cross section are shown in Figure 5.

According to geometric relationships, the strain at any location can be calculated according to Equation (19).

(19) ε=u(y+h/2−x)

In this equation, *h* is the thickness of the segment, *x* is the depth of the compression zone, and *y* is the vertical coordinate relative to the middle of the thickness with the positive direction towards the intrados.

The stress–strain laws for concrete under compression and tension can be represented by the functional relationships *σ*_c_ = *f*_c_(*ε*_c_), and the stress–strain law for the steel reinforcement is represented by *σ*_s_ = *f*_s_(*ε*_s_). Then, based on Equation (19), the stress in the concrete and the steel reinforcement at any location can be calculated by Equations (20) and (21), respectively.

(20) $$\sigma_{c}=f_{c}(\varepsilon_{c})=f_{c}(u,x,y_{c})$$

(21) $$\sigma_{s}=f_{s}(\varepsilon_{s})=f_{s}(u,x,y_{s})$$

In these equations, *y*_c_ and *y*_s_ represent the vertical coordinates of the concrete and the steel rebar.

Finally, the static equilibrium equations can be derived. Integrating the stresses across the section to determine the axial force and integrating the stress moments about the coordinate origin to determine the bending moment. The equilibrium equations are established as follows (Equations (22) and (23))

(22) $$N=\iint_{A}\sigma_{c}dA+\sigma_{ss}A_{s}+\sigma_{sc}A_{s}^{\prime }$$

(23) $$M=\iint_{A}\sigma_{c}•ydA+\sigma_{ss}A_{s}(0.5h-a_{s})+\sigma_{sc}A_{s}^{\prime }(a_{s}^{\prime }-0.5h)$$

where *A* is the area of the cross section. In the equations, *σ*_ss_ and *σ*_sc_ are the stress of the rebars at the intrados and the extrados, respectively. *A*_s_ is the area of the rebars at the intrados, and *a*_s_ corresponds to the distance from the centroid of these rebars to the inner arc surface. $A_{s}^{\prime }$ is the area of the rebars at the extrados, and $a_{s}^{\prime }$ corresponds to the distance from the centroid of these rebars to the outer arc surface.

By simultaneously solving Equations (19)–(23), the unknowns in the bending moment and axial force equilibrium equations are the curvature *u* and the compression zone height *x*. Therefore, a unique solution can be obtained. Thus, the deformation and stress state can be determined, including curvature, compression zone height, reinforcement strains and stresses, and concrete strains and stresses. The stiffness (*k*) of the segment can be obtained by dividing the bending moment *M* by the curvature *u*, as shown in Equation (24)

(24) k=M/u

## 4. Test Results

### 4.1. Material Properties

**Figure 6:**
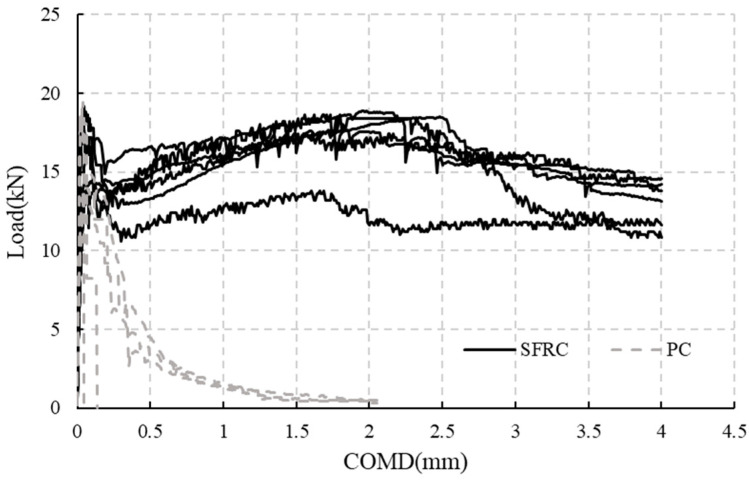
Load-CMOD curves of bending tests (6 specimens for each PC and SFRC).

The test results for the material properties are shown in Table 6 and Table 7. The load-CMOD curves of the three-point bending test according to EN 14651 [38] are shown in Figure 6. Based on the figures and tables, it is evident that SFRC and PC exhibit similar compressive and flexural tensile strengths *f*_L_, but clearly SFRC can maintain its strength after cracking, while PC experiences a rapid decrease in strength, even leading to brittle failure in some cases.

The constitutive parameters for the analytical calculation are based on these experimental results. Since direct tension and modulus tests were not conducted, the concrete tensile strength and modulus of elasticity are determined according to the Chinese Code GB 50010-2010 [48]. Specifically, the concrete tensile strength is assumed to be the value of 2.85 MPa, and the modulus of elasticity is assumed to be the recommended value of 36,000 MPa according to the Code [48]. The parameters for the analytical calculation are summarized in Table 8.

### 4.2. Segment Bending Tests

#### 4.2.1. General Analysis

**Figure 7:**
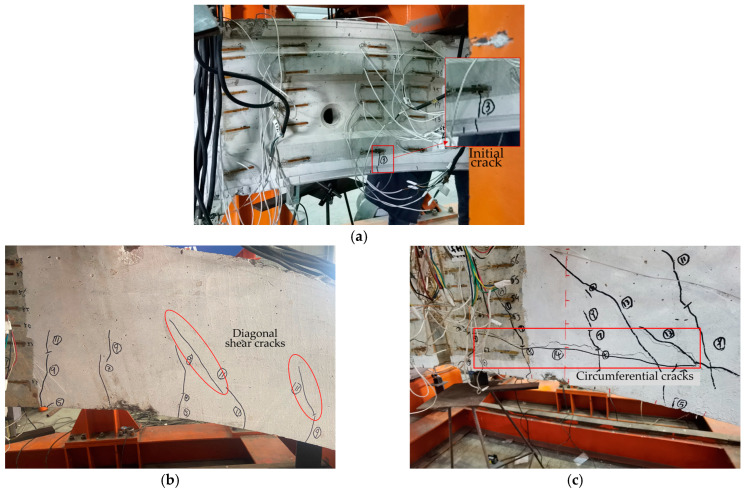

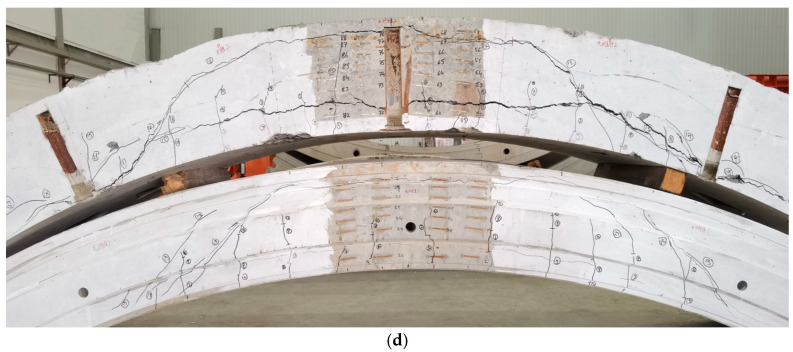
Development of cracks and segment failure: (**a**) Crack initialization; (**b**) diagonal shear cracks; (**c**) circumferential cracks; (**d**) segment failure.

For all specimens, when increasing the load, bending-tensile cracks, diagonal shear cracks, and circumferential cracks appeared sequentially, finally leading to failure (Figure 7). All phenomena were observed for both RC segments and RC-SFRC segments. The tensile cracks first developed between the loading points (Figure 7a). As the load increased, diagonal shear cracks (Figure 7b) were observed outside the loading points. Later circumferential cracks (Figure 7c) also developed, which appeared approximately 100 mm to 120 mm from the inner surface of the segment, extending circumferentially along the segment. These cracks were primarily observed at the cutting lateral surface. The occurrence of these cracks can be attributed to two factors: firstly, the test specimens were obtained by cutting single intact segments (see Section 2.1), resulting in damage to the hoop stirrup, especially for the cut side surface, secondly, the steel bars near the cut surface consisted of two adjacent rebars, complicating the consolidation of concrete near the rebars and potentially leading to localized deterioration in concrete quality. The weakened zone is near the rebars, so the zone is located circumferentially along the rebars at around 100 mm to 120 mm from the inner surface. This zone is prone to circumferential damage under large loads.

**Figure 8:**
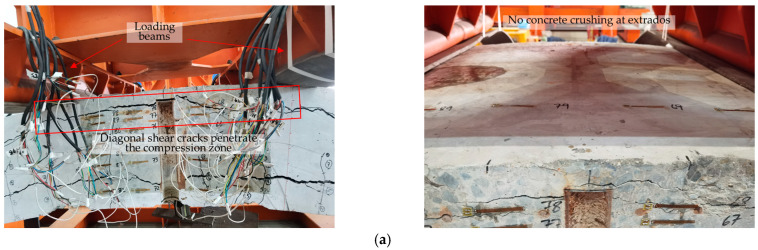

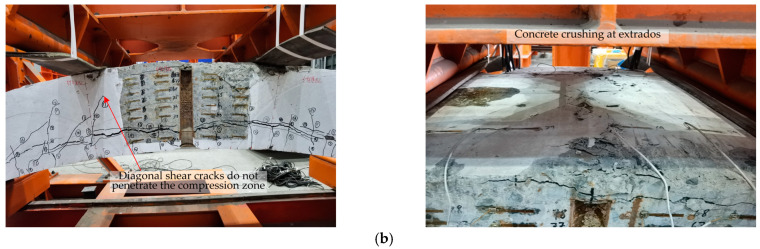
Segments failure: (**a**) The phenomena of RC segments at the ultimate state; (**b**) the phenomena of RC-SFRC segments at the ultimate state.

Due to the occurrence of circumferential cracks, the ultimate failure mode of the segments is not a typical bending failure. Segment failure resulted from the rapid propagation of circumferential cracks. However, the ultimate failure modes are different between RC segments and RC-SFRC segments (Figure 8). In the RC segments, diagonal shear cracks developed rapidly and extensively, extending into the compression zone and circumferentially penetrating. The segment was divided into three layers by the penetrating shearing cracks and circumferential cracks, thus the segment was weakened and destroyed, with no concrete crushing observed in the compression zone at the extrados. In contrast, in SFRC segments, diagonal shear crack propagation was slower and less extensive, and the cracks were smaller. Some diagonal shear cracks extended into the compression zone, but did not circumferentially penetrate on both sides of the loading points. As a result, the SFRC segments were less weakened, and sustained higher loads, with concrete crushing observed in the compression zone. This phenomenon is attributed to the ability of the steel fibers to restrain crack propagation thus enhancing the shear capacity of the segments.

Table 9 gives the loads at which various types of cracks are initiated as well as the ultimate load.

#### 4.2.2. Deflection Analysis

**Figure 9:**
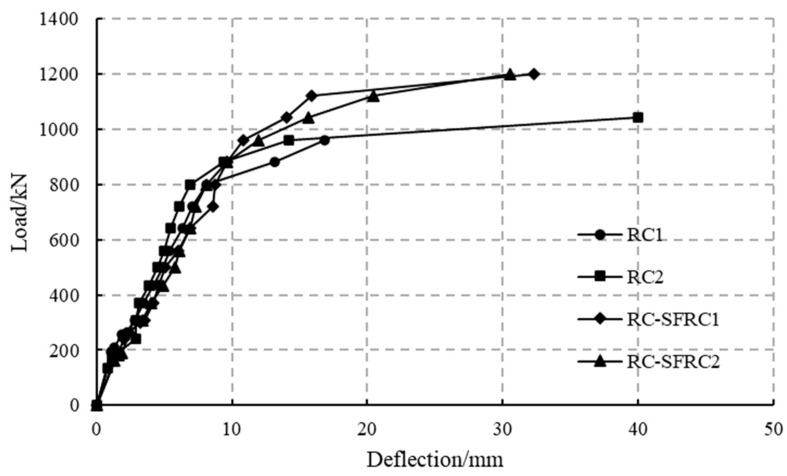
Deflection curve at mid-span.

Based on the displacement data from the LVDTs, the variation in the net deflections (D1 − (D4 + D5)/2) at mid-span with load is shown in Figure 9. From these figures and tables, it is evident that after initial cracking, the deflection increases gradually, and after the appearance of circumferential cracks, the deflection increases rapidly.

The main focus of this paper is to investigate the flexural stiffness of segments under bending loads during service conditions, where typically circumferential cracks do not occur. Therefore, the discussions in this paper primarily focus on conditions before the appearance of circumferential cracks.

#### 4.2.3. Concrete Strain Analysis

**Figure 10:**
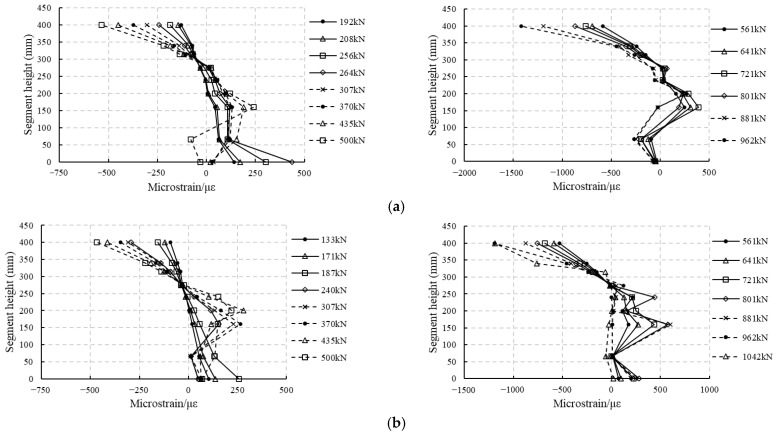
Concrete strains of RC segments at different height levels and different load values: (**a**) RCS1; (**b**) RCS2.

**Figure 11:**
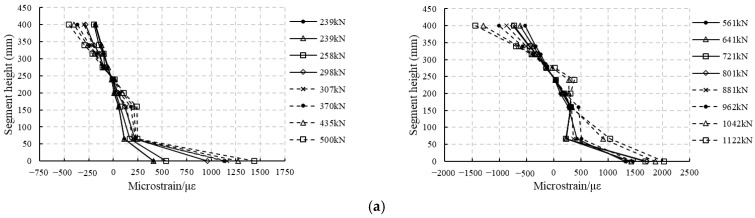

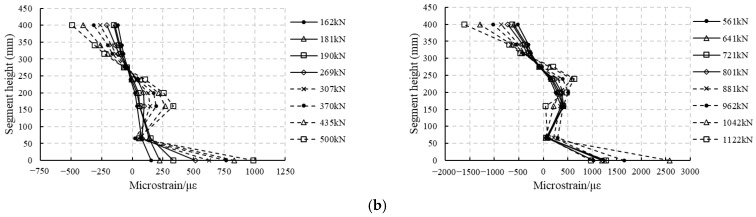
Concrete strains of RC-SFRC segments at different height levels and different load values: (**a**) RC-SFRCS1; (**b**) RC-SFRCS2.

As described in Section 2.2, concrete strains at different locations along the segment thickness were measured. Referring to Figure 3, four strain gauges were applied at nine levels on each side. The average strain measured by the eight strain gauges is used to represent the strain at that level. The results are shown in Figure 10 and Figure 11. According to these figures, the concrete strain changes linearly along the thickness before cracking. After cracking, the concrete strains are rather arbitrary in the cracked zone because of the influence of cracks, but keep changing linearly in the uncracked tensile zone and the compressive zone. In the cracked zone, the strain gauges do not cover all the concrete, and sometimes the crack occurs through the strain gauge, which makes the strain in this gauge abnormally large, sometimes adjacent to the gauge, which makes the strain in this gauge smaller than expected, resulting in arbitrary strain values. However, given the fact that the strains in the uncracked tensile zone and in the compressive zone change linearly, it can be concluded that the deformations of both RC segments and RC-SFRC segments satisfy the plane section assumption.

## 5. Analysis and Discussion

### 5.1. Comparison Between the Test Results and the Analytical Model Results

According to Figure 10 and Figure 11, the strain along the thickness changes linearly in the uncracked tensile zone and in the compressive zone. Making a linear fit of the concrete strain over the height of these zones, it can be found that the R2 (goodness of fit) is almost larger than 0.92 for RC segments (except for RCS2 at a load level of 435 kN, where R2 = 0.87), and is larger than 0.97 for RC-SFRC segments. This means the fitting is reliable. Thus, the curvature of the segment can be calculated as the reciprocal of the slope of the fitting line. The bending stiffness can then be obtained by dividing the bending moment by the curvature. At the same time, the location of the neutral axis can be obtained to be the intercept of the fitting line and the vertical axis, and the strains of the rebars can also be calculated.

**Figure 12:**
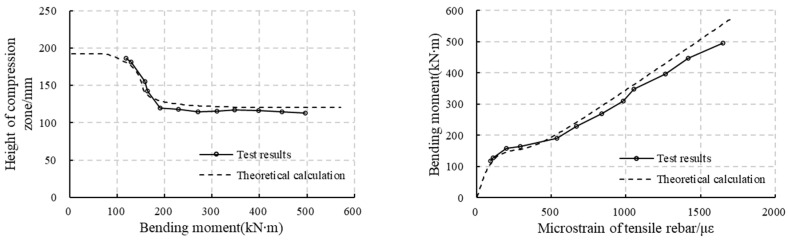

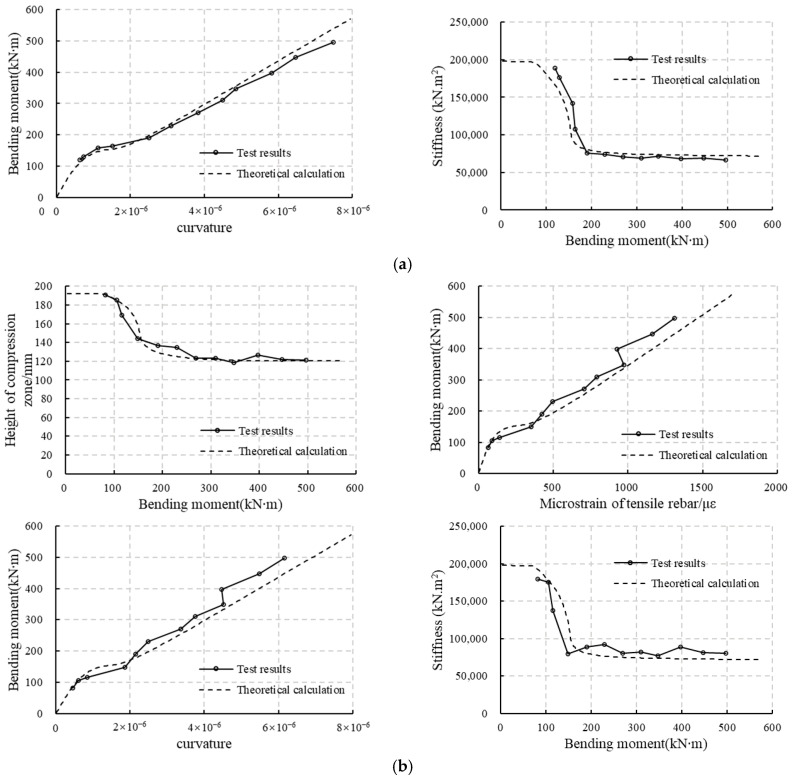
Comparison between the test results and analytical model results of RC segments: (**a**) RCS1; (**b**) RCS2.

**Figure 13:**
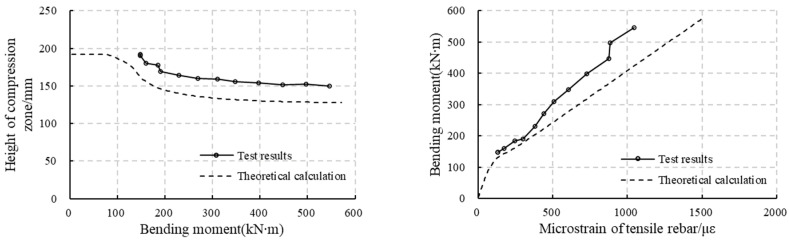

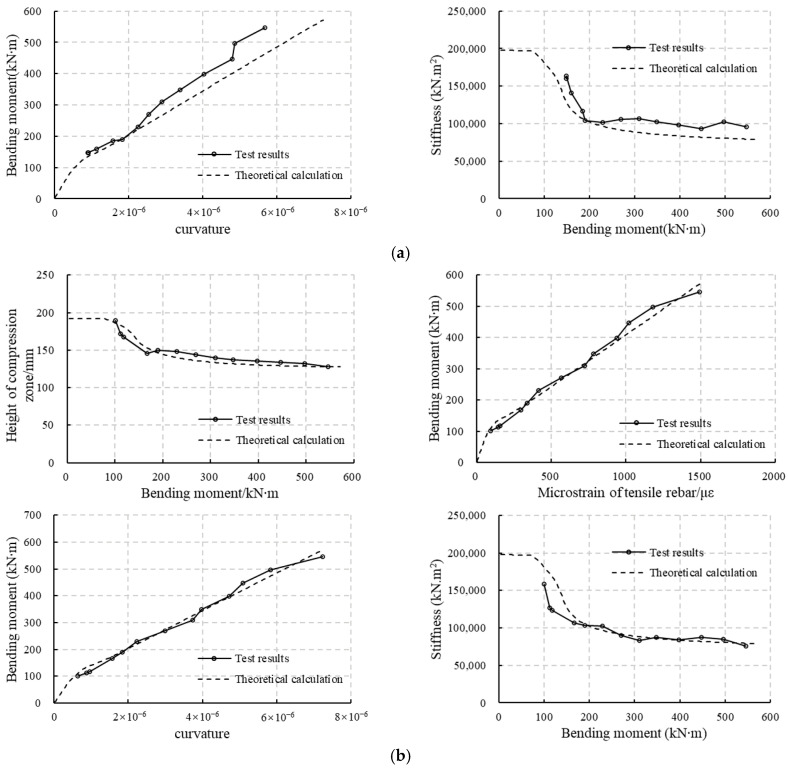
Comparison between the test results and analytical model results of RC-SFRC segments: (**a**) RC-SRFCS1; (**b**) RC-SRFCS2.

On the other hand, these values can also be calculated analytically, as explained in Section 3.3. A comparison between the test results and the results from the analytical method results is presented in Figure 12 and Figure 13. Since the ultimate failure mode is changed by the circumferential cracks, the comparison is carried out only for the stages before the circumferential cracks appear. According to the figures, except for the results of RC-SFRCS1, the analysis results and the test results match well, which means the analytical method is reliable. The relatively large discrepancy in the results of RC-SFRC1 may be caused by measurement errors of the concrete strain.

### 5.2. Bending Stiffness Variation

**Figure 14:**
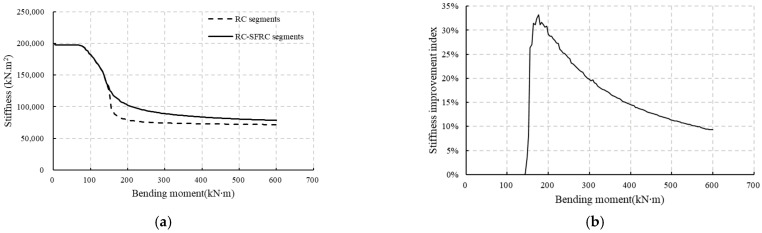
Variation in the enhancement effect of SFRC on the stiffness under the increasing bending moment obtained from the analytical analysis: (**a**) stiffnesses under increasing bending moment; (**b**) index under increasing bending moment.

According to Figure 12 and Figure 13, the compression zone and the stiffness decrease rapidly once initial cracks appear, and then become more stable, only showing a slow decrease. However, the stiffness decrease is not equal for RC segments and RC-SFRC segments, with a quicker decrease for RC segments. The ratio of the stiffness difference between RC segments and RC-SFRC segments and the stiffness of RC segments is taken as an index reflecting how much the stiffness is improved by the SFRC. This index is calculated through Equation (25), and its evolution under the increasing load is shown in Figure 14.

(25) $$P_{k}=(k_{\mathrm{SFRC}}-k_{\mathrm{RC}})/k_{\mathrm{RC}}$$

where *P*_k_ is the index of how much the stiffness is improved, and *k*_SFRC_ and *k*_RC_ are the stiffnesses of RC-SFRC segments and RC segments, respectively.

Since the test results show some scatter as shown in Figure 12 and Figure 13 due to the randomness of cracking, the index is calculated based on the results of the analysis method, as these results match well with the test results, and are also more stable. As shown in Figure 14, before the crack appears (132 kN·m), the index is 0, which means the stiffness of RC segments and RC-SFRC segments are the same, because the concrete tensile strength and elastic modulus are not influenced by fiber addition, and the rebars in the two types of segments are equal. After the crack appears, SFRC enhances the bending stiffness. However, according to the index shown in Figure 14b, the degree of improvement shows an initial rapid increase and then a gradual decrease. When the bending moment is 176 kN·m, the SFRC improvement reaches a maximum of 33%.

**Figure 15:**
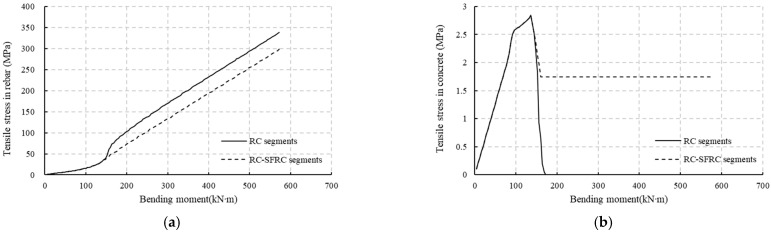
Concrete and rebar tensile stress at rebar location: (**a**) rebar tensile stress under increasing bending moment; (**b**) concrete tensile stress under increasing bending moment.

After initial cracking, it appears that, from Figure 12 and Figure 13, the height of the compression zone of the RC-SFRC segments is larger, the curvature is smaller, and consequently the stiffness is larger than that of the RC segments for qual bending moments. The fundamental factor affecting the stiffness is the material stress state. Thus, the rebar and concrete stresses at the location of the rebars for both RC segments and RC-SFRC segments are taken for comparison (Figure 15), to analyze the rationale behind the positive effect of SFRC on the stiffness after initial cracking.

As shown in Figure 15, for RC, after concrete cracking and with increasing moment, the concrete stress decreases to 0 rapidly, and the tensile force will be entirely carried by the steel rebars, causing a rapid increment in the steel rebar stress and strain, and causing a rapid curvature increment. Thus, the stiffness will decrease quickly. On the other hand, for RC-SFRC, the residual stress in the SFRC after cracking remains larger than 1.74 MPa, only a slight reduction occurs (the PC and SFRC have the same mechanical response before cracking as shown in Figure 4 considering segments OAB), and the SFRC will still bear part of the tensile force. The tensile force transferred to steel bars is limited, and the rebar strain and segment curvature will increase more slowly. Thus, the stiffness will decrease slowly. This is the mechanical reason why the stiffness of the SFRC segment is improved.

**Figure 16:**
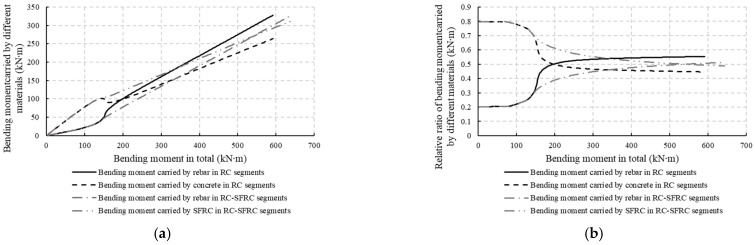
Bending moments carried by the steel rebars and concrete: (**a**) Bending moments carried by different materials; (**b**) relative ratio of bending moments carried by different materials.

Further, the bending moments carried by the steel bars and the concrete can also be determined through the analytical method described in Section 3.3. They are shown in Figure 16. It can be found that concrete carries a greater bending moment before cracking, but the difference between the bending moments carried by concrete and rebars gradually decreases after concrete cracking, and the bending moment carried by rebars can even be larger than that of concrete because the stresses in the rebars increase while the tensile stresses in concrete decrease. This is the same for both RC segments and RC-SFRC segments. This means that the rebars will play a more and more important role with increasing moments, while the concrete plays a lesser role. Thus, the improvement of stiffness by SFRC will decrease with the increase in load.

The previous analysis makes it clear why the improvement of stiffness caused by SFRC initially increases and subsequently decreases. In the initial cracking stage, the stress in the PC drops rapidly to zero after cracking, causing the steel bars in RC segments to bear more load. Consequently, the strain increases rapidly, leading to a rapid increase in segment curvature and a rapid decrease in stiffness. However, for SFRC, because the stress decreases less after cracking, the segment curvature increases more slowly, and the stiffness decreases more slowly. Therefore, compared to RC segments, the stiffness of RC-SFRC segments is enhanced. In the later loading stages, as the bending moment borne by the steel bars increases, the role of concrete in bearing the load diminishes, and thus the stiffness improving effect of SFRC gradually decreases.

## 6. Conclusions

The influence and mechanical mechanism of SFRC on the bending stiffness of tunnel segments subjected to bending moments are studied by full-scale tests and analytical calculations. The following conclusions can be drawn.

1. The derived analytical method to calculate the stiffness of the SFRC segment by considering the SFRC’s residual strength is verified to be reliable in the SLS through comparison with experimental data.

2. The research results fully confirm that the use of SFRC can improve the segment stiffness in serviceability conditions.

3. The residual stress of SFRC which occurs after cracking is the main reason for the improvement of the bending stiffness, because it can reduce the increase in rebar stresses, and delay the deformation of the RC-SFRC segment.

4. The improving effect of SFRC on the bending stiffness of segments first becomes stronger but then becomes weaker as the load increases.

## Figures and Tables

**Table 1:** Summary of research results for SFRC segments from the literature.

	Reinforcement Ratio (%)	Reinforcement Ratio after Rebar Replacement (%)	Fiber Content (%)	SFRC Class *	Improvement of Bearing Capacity when Crack Width is 0.2 mm (%)	Improvement of Ultimate Bearing Capacity (%)	Improvement of Stiffness ** (%)	Reference
SFRC segments	0.15	0	0.47	6.5 c	+40	−18	+	[17]
	Unkn ***	0	1.5	Unkn	+98	−66	Unkn	[18]
	Unkn	0	0.51	4.0 g	Unkn	Unkn	Unkn	[19]
	Unkn	0	0.64	4.0 d	Unkn	Unkn	Unkn	[20]
RC-SFRC segments with rebar partially replaced	0.67	0.45	0.45	Unkn	+38	+48	Unkn	[21]
	0.67	0.3	0.45	Unkn	+3	+21	Unkn	
	0.257	0.21	0.38	3.5 c	−3.9	+1.7	+190.6	[1]
	0.257	0.195	0.51	4.0 c	+2.6	+1.7	+129.7	
	0.22	0.18	1.1	2.0 e	+56	+55	Unkn	[5]
	0.22	0.13	1.1	2.0 e	+	=	Unkn	[6]
	Unkn	0.24	0.51	4.0 g	Unkn	Unkn	Unkn	[19]
	0.32	0.13	0.66	1.0 c	−30	−45	-	[7]
RC-SFRC segments without rebar replaced	0.257	0.257	0.38	3.5 c	+2.6	+13.9	+128.1	[1]
	0.257	0.257	0.51	4.0 c	+26.2	+15.6	+217.2	

* SFRC class is used to represent the mechanical properties of SFRC, and the classification method is specified in *fib* Model Code [22,23]. ** All improvements relate to RC segments. *** “Unkn” indicates information not provided in the literature.

**Table 2:** Concrete mixture composition (kg/m^3^).

	Cement (P.O 52.5)	Fly Ash (class I)	Water	Sand	Coarse Aggregate	Superplasticizer	Steel Fibers
PC	370	90	148	702	1132	5.52	0
SFRC	370	90	148	723	1112	7.21	30

**Table 3:** Characteristics of the steel fibers.

Sample	Type	Yield Strength (MPa)	Length (mm)	Diameter (mm)	Length/Diameter (-)
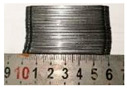 Unit: mm	Hook-end fibers	1700	60	0.75	80

**Table 4:** Size and number of concrete specimens for testing of material properties.

Test Item	Concrete Type	Size (mm)	Number
Compression strength (compressive test [37])	PC	150 × 150 × 150 (cube)	3
	SFRC		3
	PC	150 × 150 × 300 (prismoid)	3
	SFRC		3
Flexural tensile strength (three-point bending test [38])	PC	150 × 150 × 550 (beam)	6
	SFRC		6

**Table 5:** Values of the loading steps.

Step	Load (kN)	Step	Load (kN)
1	192	10	641
2	208	11	721
3	256	12	801
4	264	13	881
5	307	14	962
6	370	15	1042
7	435	16	1122
8	500	17	1202
9	561	18	1282

**Table 6:** Test results for compressive strength (MPa).

	Compressive Strength of Cubic Specimens			Compressive Strength of Prismatic Specimens		
	Mean Value	STDV	COV	Mean Value	STDV	COV
PC	60.60	2.45	0.04	56.03	2.27	0.04
SFRC	60.43	1.89	0.03	48.43	1.65	0.03

**Table 7:** Test results for flexural tensile strength (MPa).

		*f*_L_	*f*_R1_	*f*_R2_	*f*_R3_	*f*_R4_
PC	Mean value	5.38	-	-	-	-
	STDV	1.09	-	-	-	-
	COV	0.20	-	-	-	-
SFRC	Mean value	5.22	4.73	5.42	5.29	4.60
	STDV	0.86	0.52	0.54	0.69	0.38
	COV	0.16	0.11	0.10	0.14	0.08

**Table 8:** Parameters for the constitutive model.

	Tension				Compression				
	*E*_c_	*f*_ct_	*f*_R1_	*f*_R3_	*f*_fcu_	*f*_cf,r_	*V*_f_	*l*_f_	*d*_f_
PC	36,000	2.85	-	-	60	56.03	-	-	-
SFRC	36,000	2.85	4.73	5.29	60	48.43	0.385%	60	0.75

**Table 9:** Typical load values (kN).

	Crack Initialization	Shear Crack Appearance	Circumferential Crack Appearance	Ultimate Load
RC1	192	721	881	960
RC2	187	881	881	1042
RC-SFRC1	239	962	1042	1202
RC-SFRC2	190	881	962	1202

## Data Availability

The original contributions presented in this study are included in this article, and further inquiries can be directed to the corresponding author.
